# Refractive outcomes of table-mounted and hand-held auto-refractometers in children: an observational cross-sectional study

**DOI:** 10.1186/s12886-021-02199-5

**Published:** 2021-12-09

**Authors:** Müjdat Karabulut, Sinem Karabulut, Aylin Karalezli

**Affiliations:** Department of Ophthalmology, Mugla Sıtkı Koçman University Medical School, 48300 Mugla, Turkey

**Keywords:** Autorefractors, Cylindrical power, Jackson cross-cylinder, Spherical equivalent spherical power

## Abstract

**Background:**

To compare the refractive results of hand-held and table-mounted autorefractors.

**Methods:**

We designed this study as an observational, cross-sectional study. We compared the mean spheric and cylinder power, spherical equivalent, Jackson cross-cylinder values, determined the limits of agreement (LoA), and evaluated the reliability of two autorefractors.

**Results:**

We evaluated 256 eyes of 256 pediatric patients (mean age, 9.12 ± 2.26 years; range, 5–16 years). 49% of the patients were female, and 51% were male. The Nidek HandyRef-K autorefractor measured relatively more astigmatism (*P* < 0.001) and less hyperopia (*P* = 0.024). The mean differences and 95% LoA were 0.06 D ± 0.47 D (− 0.82 D to 0.98 D) in spherical power, 0.08 D ± 0.28 D (− 0.47 D to 0.64 D) in cylindrical power, 0.11 D ± 0.47 D (− 0.81 D to 1.01 D) in spherical equivalent, 0.02 D ± 0.36 D (− 0.73 D to 0.69 D) in Jackson cross-cylinder power at 0°, 0.005 D ± 0.54 D (− 1.07 D to 1.06 D) in Jackson cross-cylinder power at 45°_._ We found the difference within 0.50 D in 244 (95%) eyes for spherical power, in 245 (96%) eyes for cylindrical power, 228 (89%) eyes for spherical equivalent, 224 (87%) eyes for Jackson cross-cylinder power at 0°, 213 (83%) eyes for Jackson cross-cylinder power at 45°_._ When comparing devices, there were strong correlations for spherical power (Spearman’s rho = 0.99, *P* < 0.001), cylindrical power (Spearman’s rho = 0.88, *P* < 0.001), and spherical equivalent (Spearman’s rho = 0.98, *P* < 0.001).

**Conclusion:**

Two autorefractors showed clinically applicable agreement limits; excellent reliability for spherical power and spherical equivalent and good reliability for cylindrical power; high positive percent agreement for spherical and cylindrical power, spherical equivalent, Jackson cross-cylinder power at 0°and 45°. These results showed that both devices might be used interchangeably for screening of refractive error in children.

## Introduction

Amblyopia is a unilateral or, rarely, bilateral reduction of best-corrected visual acuity that cannot be attributed directly to the effect of any structural abnormality of the eye or visual pathways [1].. It is also a neurodevelopmental disorder associated with the visual cortex and lateral geniculate nucleus abnormalities [2, 3]. Refractive amblyopia is a type of amblyopia and consists of ametropic, meridional, and anisometropic subtypes [4]. Ametropic amblyopia may arise from bilateral 5.0–6.0 Diopter (D) or more myopia and 4.0–5.0 D or more hyperopia. Meridional amblyopia may happen in the presence of 2.0–3.0 D or more astigmatism. Anisometropic amblyopia may arise from anisomyopia (3.0–4.0 D or more), anisoastigmatism (2.0 D or more), and anisohyperopia (1.0–1.5 D or more) [5]. Therefore, timely identification of refractive errors in children is crucial for preventing refractive amblyopia. For this purpose, national pediatric vision screening programs have been planned and may vary among countries depending on the country’s income [6]. Cycloplegic retinoscopy is the gold standard for evaluating refractive errors in children because refractive error can be obtained objectively by completely relaxing accommodation with this method [7, 8]. However, it is time-consuming and requires an experienced clinician.

The autorefractors have an essential role in preventing the development of refractive amblyopia by accurately screening the amblyogenic refractive errors.. Various techniques, such as hand-held and table-mounted auto-refractometers, are commonly used to detect refractive errors [9]. Although these devices rapidly measure the refractive errors and provide valid results, they are bulky,non-portable, and not appropriate for immobile patients [10].

On the other hand, hand-held auto-refractometers are small, portable, and can be used anywhere as needed. They are also practical and appropriate for newborns, infants, and bedridden patients or those with reduced mobility restricting their sitting ability.

Reliability determines the consistency or correlation of two values measured with different people or the same person at different times [11]. If the two devices give reliable results, they can be used interchangeably.

In this cross-sectional study, we compared the cycloplegic measurements of a table-mounted (Topcon TRK-2P; Topcon Medical Systems, Inc., Tokyo, Japan) and hand-held (Nidek HandyRef-K; Nidek Co., Ltd., Tokyo, Japan) auto-refractometer, and determined the limits of agreement (LoA) and reliability of both devices.

## Methods

Pediatric patients who visited the ophthalmology clinic for regular ocular examination were enrolled in this observational cross-sectional study. We included the children, aged 5 to 16 years, who have no history of ocular surgery (Corneal, lenticular, or retinal surgery), sensitivity to cyclopentolate, epilepsy, and were able to cooperate enough with the measurements to gather reliable results. We excluded those patients with manifest strabismus or motility disorders; nystagmus; media opacity; congenital or acquired corneal, lenticular, retinal, choroidal, or optic disc abnormalities; and participants who were unable to cooperate with the measurements. After informing the patients and their parents or legal representatives, the authors obtained consent from children, parents, or legal representatives. All patients underwent comprehensive ocular examination, including visual acuity, anteroposterior segments check, ocular motility, and the cover-uncover test. Cyclopentolate 1% (Cycloplegin; Abdi Ibrahim, Istanbul, Turkey) was applied three times at intervals of 5 min. Patients waited for about 45 min to attain complete cycloplegia and dilated pupils that did not react to intense light. The evaluations were performed randomly in the same room and light condition, with the Topcon TRK-2P and Nidek HandyRef-K devices operated by a single expert blinded to the study. This expert was a trained professional with 9 years of experience in a clinical setting and did not know the participants’ personal information and study’s name, purpose, and design until the study was over.

Measurement accuracy check was performed daily with a 0.12 D model eye for both devices before the evaluations. Since the measured results (0.12 D) did not differ from the values indicated on the model eye, the devices were not calibrated. Additionally, the same devices were used throughout the study.

The Nidek HandyRef-K is a closed-field hand-held, portable, easy-to-use, monocular auto-refractometer that detects refractive errors in infants, any age of childhood, and adolescents sitting, standing, or in a supine position. A fogging mechanism is exerted to reduce accommodation. Its measurement range is − 20.00 D to + 20.00 D sphere (0.12 D/0.25 D increments), cylinder 0 D to 12.00 D (0.12 D/0.25 D increments), and axis 0° to 180° (1°/5° increments) [12].

Topcon TRK-2P is a table-mounted instrument that assembles a refractor keratometer, non-contact tonometer, and pachymeter in one device. However, these devices are large, difficult to move, and not appropriate for bedridden patients, infants, or any patient who cannot sit down to have measurements taken. The refractive measurement range of Topcon TRK-2P is − 30 D to + 25 D sphere (0.12 D/0.25 D increments), 0 D to 12 D cylinder (0.12 D/0.25 D increments), and 0° to 180° (1°/5° increments) astigmatic axis [13]. Topcon TRK-2P also uses a fogging mechanism to diminish accommodation.

The standard refractometer model was used for both devices. We averaged three consecutive, valid cycloplegic measurements of spherical power (S_pwr_), cylindrical power (C_pwr_), and cylindrical axis (C_ax_) for each device. We analyzed average values in the Statistical Package for the Social Sciences (SPSS) version 21.0.0.0. If three consecutive measurements from each device differed by more than 0.50 D, repeated evaluations were done until the variations decreased below 0.50 D to get valid results.

The spherical equivalent (SE) and Jackson cross-cylinder power at 0° (J_0_) and 45°(J_45_) axis were computed using the following formulas: SE = S_pwr_ + C_pwr_/2; J_0_ = -(C_pwr_/2) cos 2C_ax_; and J_45_ = -(C_pwr_/2) sin 2C_ax_, respectively. Because the refractive errors of two eyes are correlated, measurements of the left eyes were analyzed.

All subjects were divided into subgroups according to the mean S_pwr_ and C_pwr_ of the Topcon TRK-2P values. The subgroups were designed considering the American Academy of Ophthalmology guidelines for correcting more than − 3.00 D and + 4.50 D isoametropia, − 3.00 D and + 1.50 D anisometropia, 2.00 D astigmatic refractive error in young children to prevent the development of refractive amblyopia [5]. This guideline was used only for classification into subgroups. Although our participants’ age ranged from 5 to 16 years, we wanted to compare the measurement of the two devices and define the differences in these amblyogenic refractive errors. We also compared the mean astigmatic refractive error under 1.00 D since it is mostly seen in clinical practice.

The positive percent agreement (PPA) is a proportion of individuals with the target condition by the imperfect reference standard who test positive. It can be used to determine the accuracy of two devices in the absence of the gold standard [14]. We calculated PPA within 0.5 D by estimating the proportion of difference within 0.5 D for all parameters.

After testing the normality and homogeneity of variables with the Shapiro-Wilk, Kolmogorov-Smirnov and Levene’s tests (*p* < 0.05 for all variables with all tests), the Wilcoxon signed-rank test was performed. The Bland-Altman plot was generated to determine the 95% LoA. Spearman’s rank correlation coefficient was used to assess reliability. Spearman’s rank correlation coefficient equal to or greater than 0.9 and between 0.8 and 0.9 demonstrated excellent and good reliability. *P* < 0.05 was respected as statistically significant.

## Results

**Fig. 1:**
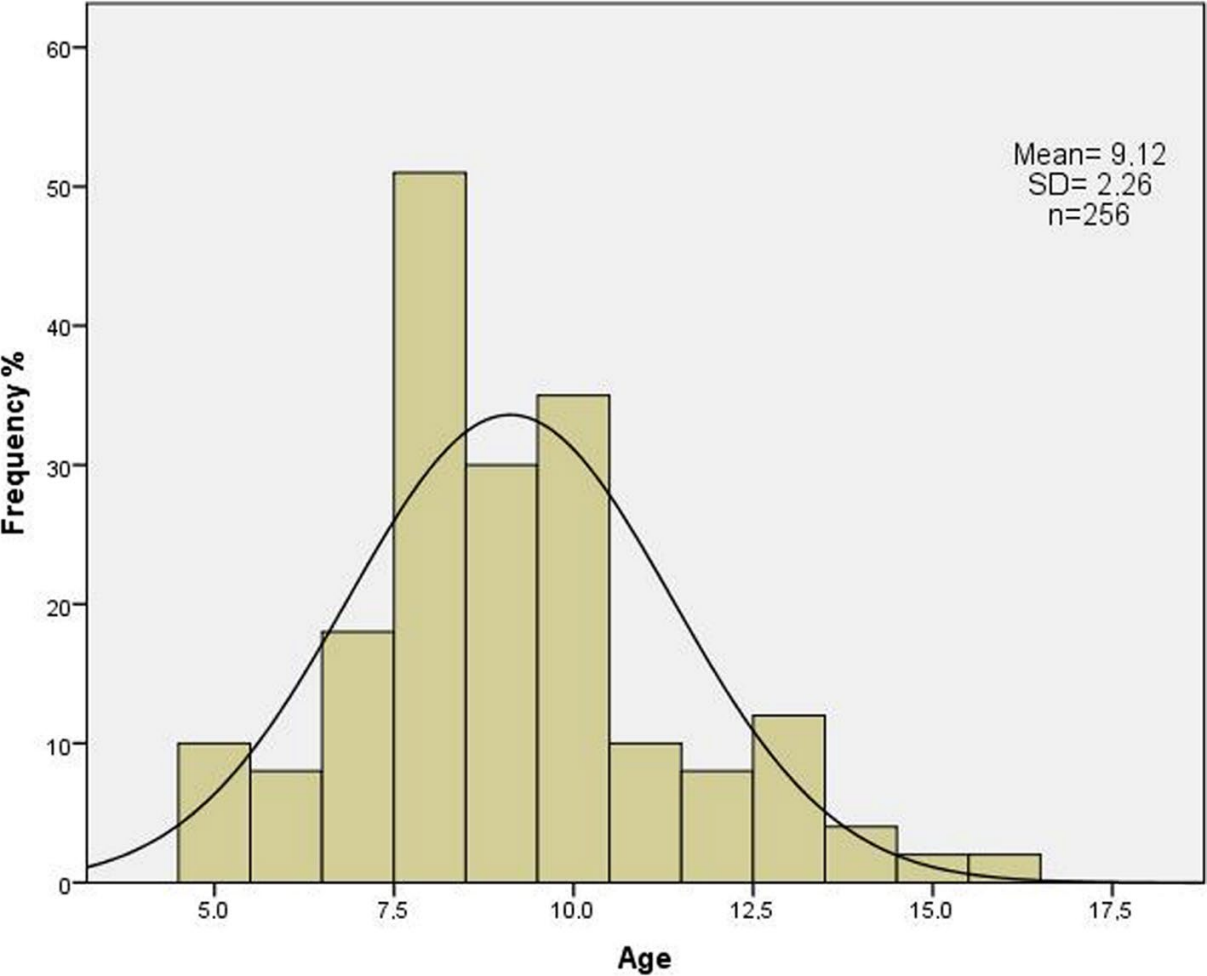
The age (years) distribution of the enrolled patients (*n* = 256)

Two hundred seventy patients were enrolled, and 14 of them were excluded from the study due to exclusion criteria (Eight invalid results, five manifest strabismus, one choroidal coloboma). The left eyes of 256 Caucasian pediatric patients were evaluated in this study. The gender distribution was 127 females (49%) and 129 males (51%). Sixty-nine (26.9%) of the patients had a type of refractive amblyopia (29 [11.3%] ametropic, 26 [10.2%] anisometropic, 14 [5.5%] meridional amblyopia) when they enrolled in the study. The mean age (± standard deviation [SD]) was 9.12 ± 2.26 years (range, 5–16 years). Figure 1 shows the age distribution.

When comparing the two devices, there were no significant differences in S_pwr_, J_0_, or J_45_ (*P* = 0.191, *P* = 0.560, *P* = 0.247, respectively) (Table 1). However, compared to Topcon TRK-2P, the Nidek HandyRef-K autorefractor measured more astigmatism (mean C_pwr_, *P* < 0.001), less hyperopia (SE, *P* = 0.024) regarding the mean SE, and significantly bigger C_ax_ (*P* = 0.037) (Table 1).

**Table 1:** Comparison of the refractive measurement of two devices in all eyes

	Topcon TRK-2P	Nidek Handy Ref-K	*P*-value
S_pwr_ (D) Mean ± SD	2.23 ± 3.45	2.17 ± 3.39	0.191
Range	(− 4.50)-(15.25)	(− 4.75)-(14.75)	
C_pwr_ (D) Mean ± SD	− 0.75 ± 0.83	−0.84 ± 0.85	**< 0.001**
Range	(−4.75)-(0.00)	(− 5.00)-(0.00)	
C_ax_ (^0^) Mean ± SD	82.84 ± 72.94	100.38 ± 74.42	**0.037**
Range	0–180	0–180	
SE (D) Mean ± SD	1.85 ± 3.35	1.75 ± 3.30	**0.024**
Range	(−4.88)-(14.88)	(−5.25)-(14.50)	
J_0_ (D) Mean ± SD	− 0.08 ± 0.36	−0.06 ± 0.36	0.560
Range	(− 1.99)-(1.80)	(− 1.57)-(1.90)	
J_45_ (D) Mean ± SD	−0.05 ± 0.42	−0.04 ± 0.47	0.247
Range	(− 2.16)-(1.54)	(− 1.92)-(2.40)	

*S*_*pwr*_ Spherical power, *C*_*pwr*_ Cylindrical power, *C*_*ax*_ Cylindrical axes, *SE* Spherical equivalent, *SD* Standard deviation, *D* Diopter, *J*_*0*_ Jackson cross-cylinder power at 0° axis, *J*_*45*_ Jackson cross-cylinder power at 45° axis

**Fig. 2:**
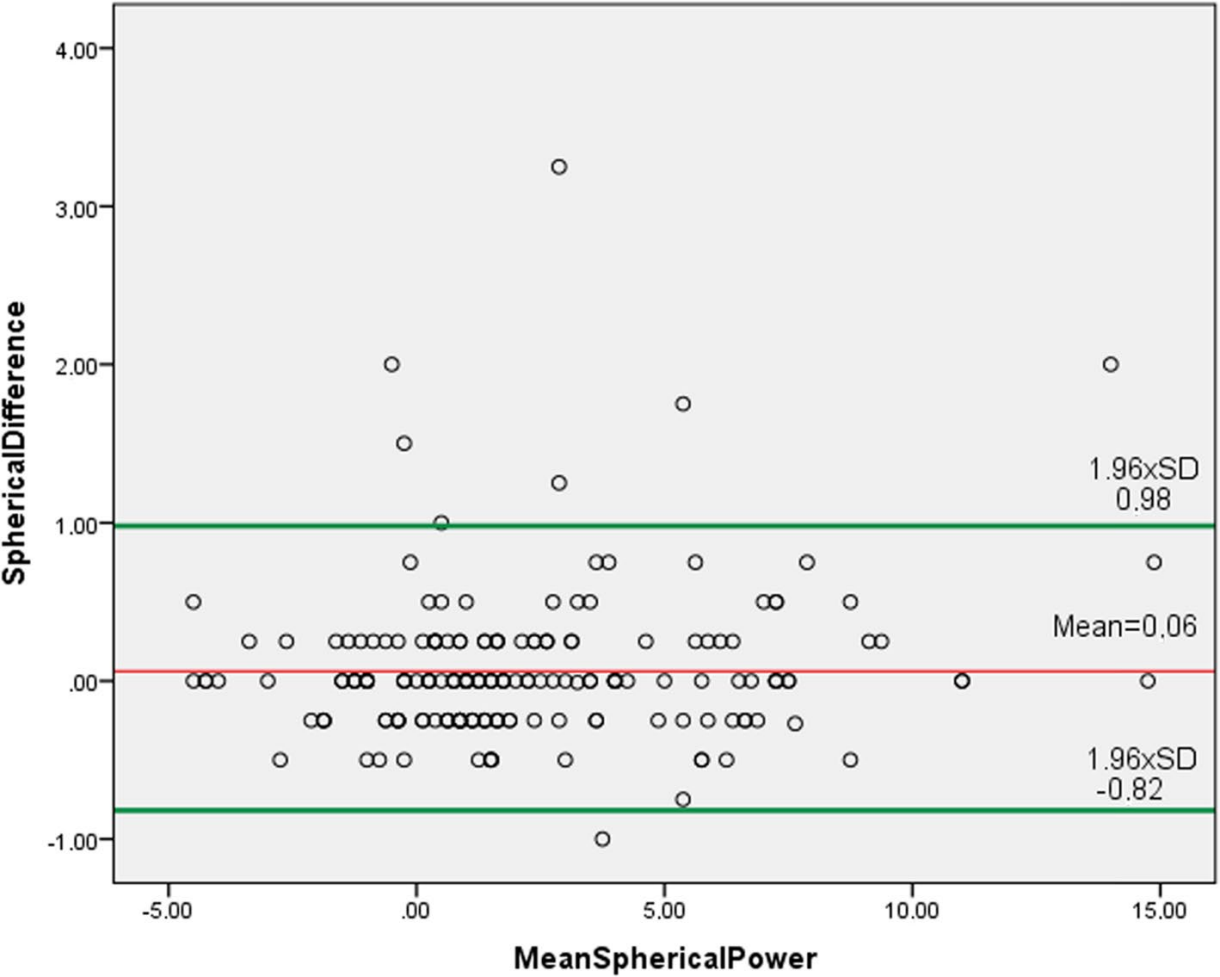
Bland Altman plot showing the agreement between Topcon TRK-2P and Nidek HandyRef-K for the mean spherical power. The middle line demonstrates the mean difference of spherical power (0.06 D ± 0.47 D), and the other two side lines show the 95% limits of agreement (− 0.82 D to 0.98 D)

**Fig. 3:**
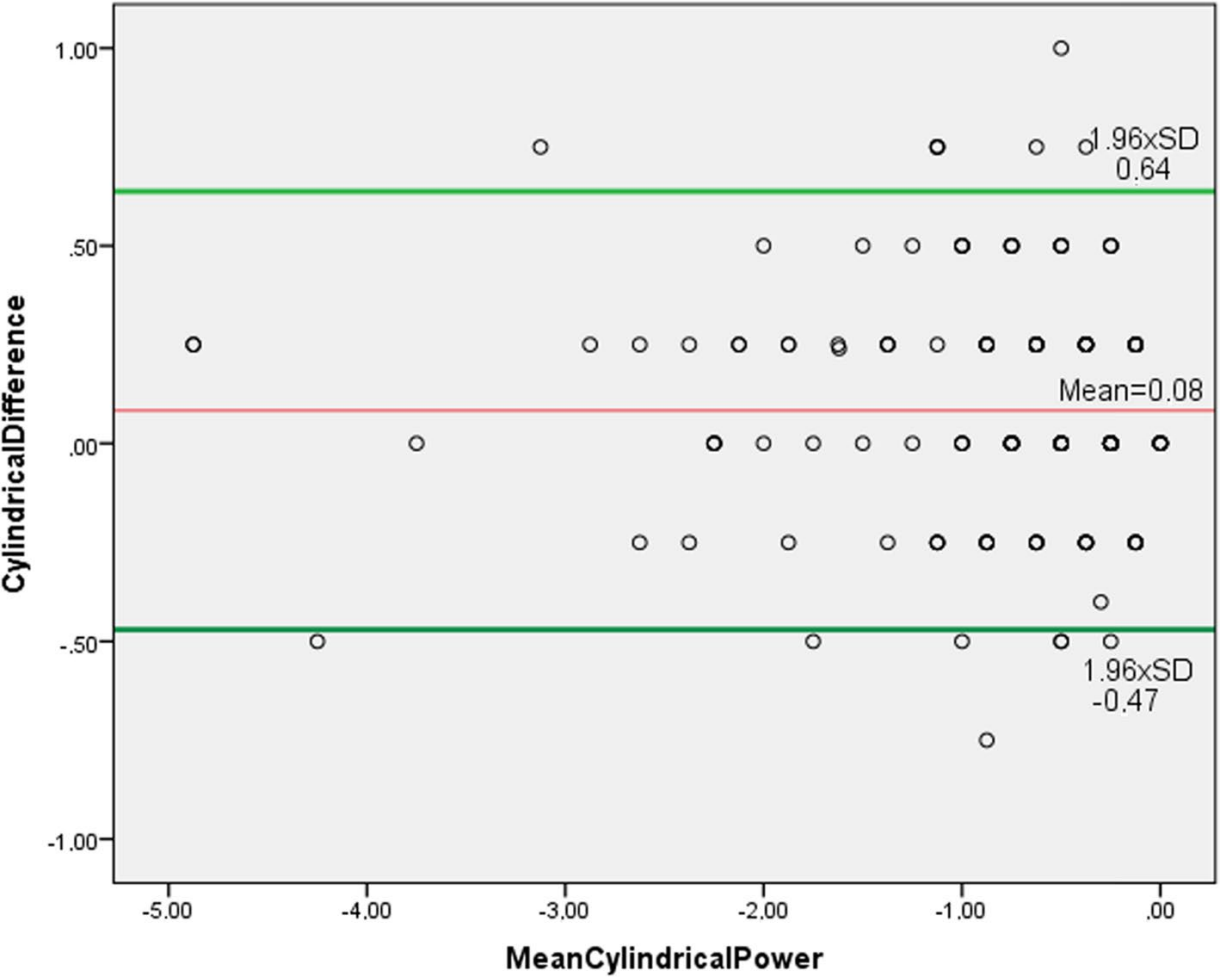
Bland Altman plot showing the agreement between Topcon TRK-2P and Nidek HandyRef-K for the mean cylindrical power. The middle line demonstrates the mean difference (0.08 D ± 0.28 D), and the other two side lines show the 95% limits of agreement (− 0.47 D to 0.64 D)

**Fig. 4:**
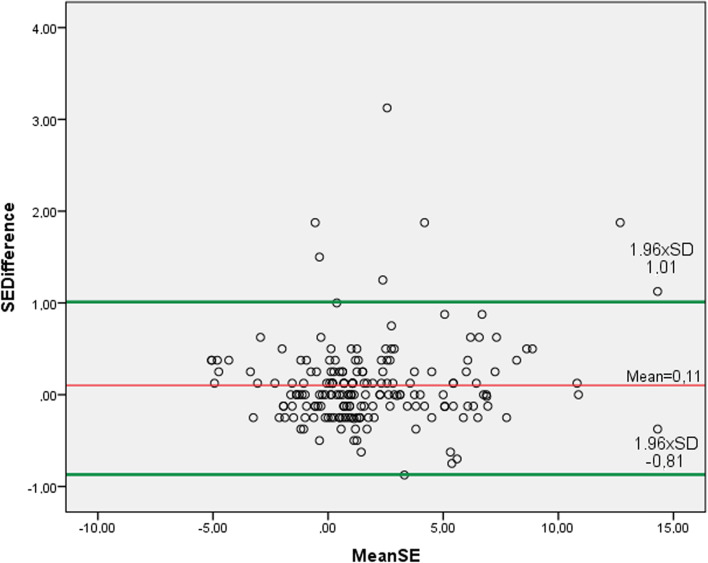
Bland Altman plot showing the agreement between Topcon TRK-2P and Nidek HandyRef-K for the mean spherical equivalent. The middle line demonstrates the mean difference (0.11 D ± 0.47 D), and the other two side lines show the 95% limits of agreement (− 0.81 D to 1.01 D)

**Fig. 5:**
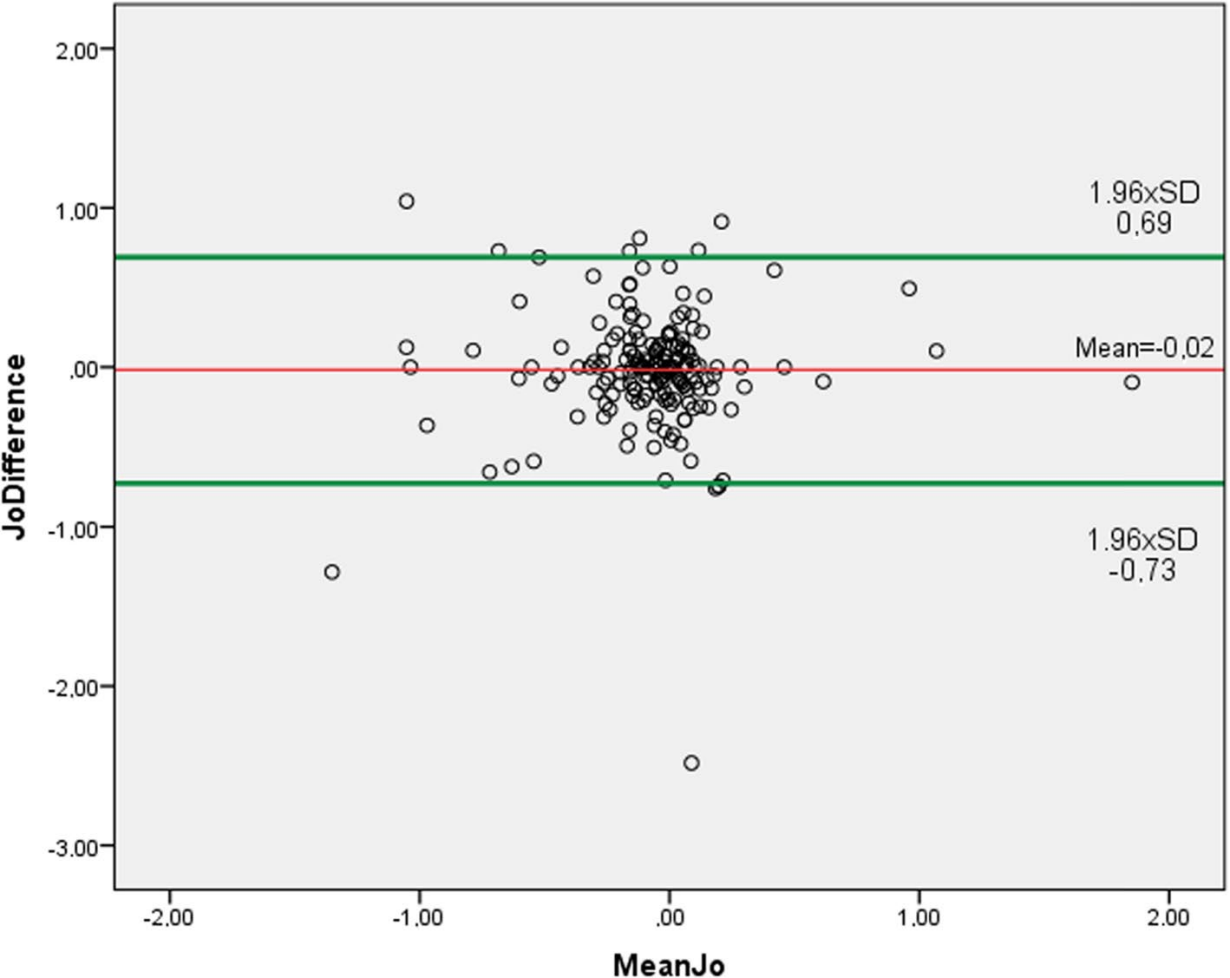
Bland Altman plot showing the agreement between Topcon TRK-2P and Nidek HandyRef-K for the mean Jackson cross-cylinder power at 0°. The middle line demonstrates the mean difference (0.02 D ± 0.36 D), and the other two side lines show the 95% limits of agreement (− 0.73 D to 0.69 D)

**Fig. 6:**
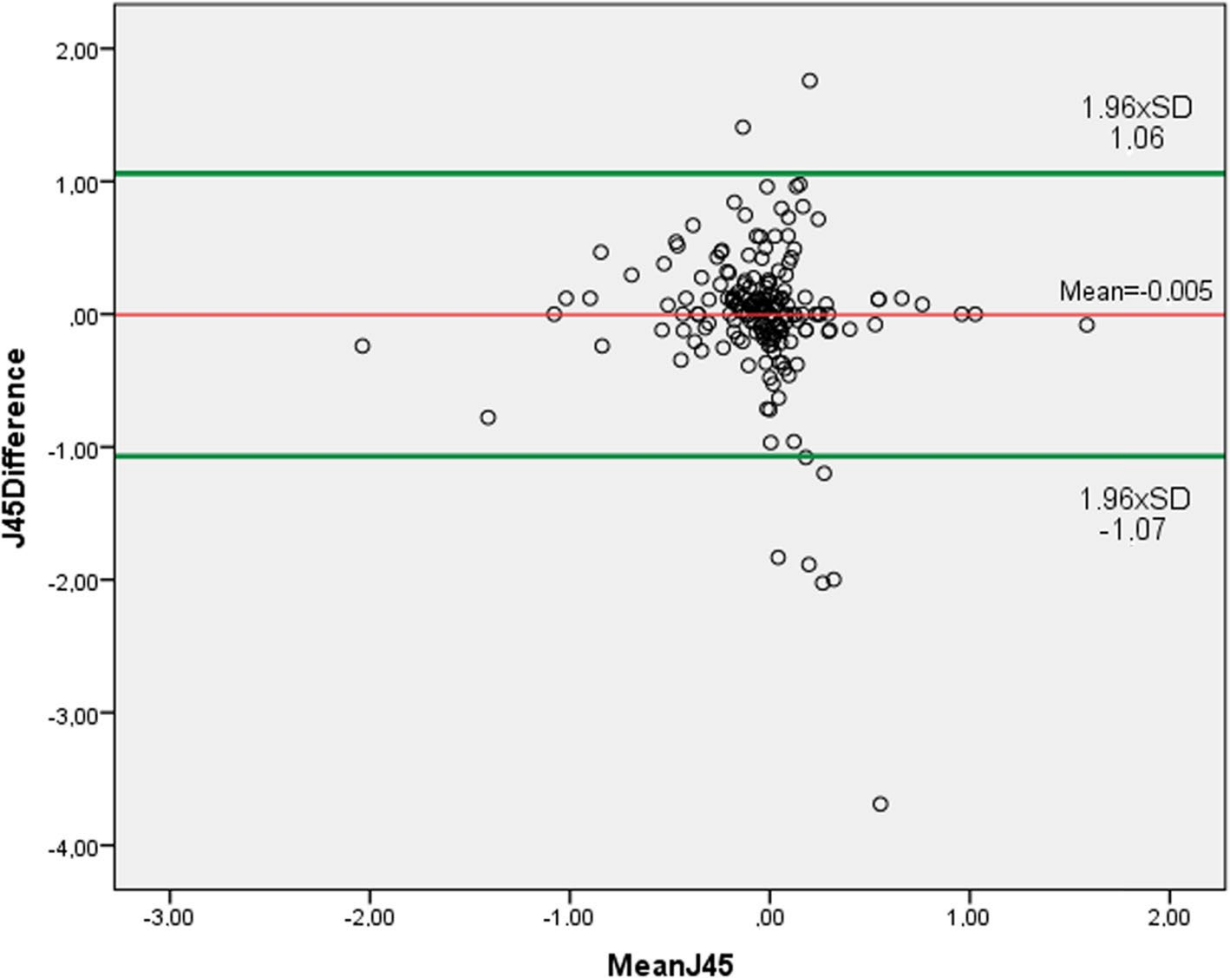
Bland Altman plot showing the agreement between Topcon TRK-2P and Nidek HandyRef-K for the mean Jackson cross-cylinder power at 45°. The middle line demonstrates the mean difference (0.005 D ± 0.54 D), and the other two side lines show the 95% limits of (− 1.07 D to 1.06 D)

The mean differences and 95% LoA were: for S_pwr_, 0.06 D ± 0.47 D (− 0.82 D to 0.98 D) (Fig. 2); for C_pwr_, 0.08 D ± 0.28 D (− 0.47 D to 0.64 D) (Fig. 3); for SE, 0.11 D ± 0.47 D (− 0.81 D to 1.01 D) (Fig. 4); for J_0_ 0.02 D ± 0.36 D (− 0.73 D to 0.69 D) (Fig. 5); and for J_45_ 0.005 D ± 0.54 D (− 1.07 D to 1.06 D) (Fig. 6)_._ We found the difference within 0.50 D in 244 (95%) eyes for S_pwr_, in 245 (96%) eyes for C_pwr_, 228 (89%) eyes for SE, 224 (87%) eyes for J_0_, 213 (83%) eyes for J_45._

When comparing the two devices, there was a strong correlation for S_pwr_ (Spearman’s rho = 0.99, *P* < 0.001), C_pwr_ (Spearman’s rho = 0.88, *P* < 0.001), SE (Spearman’s rho = 0.98, *P* < 0.001); a moderate positive correlation for J_0_ (Spearman’s rho = 0.32, *P* < .001); and a weak positive correlation for J_45_ (Spearman’s rho = 0.17, *P* = 0.018) (Table 2).

**Table 2:** The reliability of two devices for S_pwr_, C_pwr_, SE, J_0_, and J_45_ with Spearman’s correlation coefficient

	S_**pwr**_	C_**pwr**_	SE	J_**0**_	J_**45**_
**Rho**	0.99	0.88	0.98	0.32	0.17
***p*-value**	**< 0.001**	**< 0.001**	**< 0.001**	**< 0.001**	**0.018**

*S*_*pwr*_ Spherical power, *C*_*pwr*_ Cylindrical power, *SE* Spherical equivalent, *J*_*0*_ Jackson cross-cylinder power at 0° axis, *J*_*45*_ Jackson cross-cylinder power at 45° axis, *Rho* Spearman’s rho

In our subgroup analyses, compared to the Topcon TRK-2P, the Nidek HandyRef-K device showed significantly less hyperopia in two subgroups: those with S_pwr_ values between + 1.50 D and + 4.50 D and those with S_pwr_ values more than + 4.50 D (*P* = 0.031 and 0.045, respectively). Also, compared to the Topcon TRK-2P, the Nidek HandyRef-K device showed more myopia in the myopia subgroup with S_pwr_ values of more than − 3.00 D (*P* = 0.026, Table 3).

**Table 3:** Comparison of the mean S_pwr_ of two devices in the subgroups for S_pwr_

Subgroup for S_pwr_	Age (Year)	N	Topcon TRK-2P	Nidek Handy Ref-K	*P*-value
	Mean (Range)		Mean ± SD		
S_pwr_ ≥ − 3.00D	11.11(6–14)	24	−4,19 ± 0.77	− 4.40 ± 0.90	**0.026**
0.00D ≤ S_pwr_ < − 3.00D	7.98(5–15)	76	−1.14 ± 0.45	− 1,18 ± 0.38	0.541
0.00 ≤ S_pwr_ < + 1.50D	8.22 (5–16)	54	0.62 ± 0.53	0.58 ± 0.78	0.305
+ 1.50D ≤ S_pwr_ < + 4.50D	7 (5–13)	61	2.01 ± 1.02	1.87 ± 1.06	**0.031**
S_pwr_ ≥ + 4.50D	6.12 (5–13)	41	6.75 ± 2.64	6.51 ± 2.61	**0.045**

*S*_*pwr*_ Spherical power, *SD* Standard deviation, *D* Diopter, *N* Number

Compared to the Topcon TRK-2P, in the subgroup with C_pwr_ less than − 1.00 D, the Nidek HandyRef-K device also detected more C_pwr_ and significantly different C_ax_ values (*P* < 0.001, *P* = 0.025, respectively; Table 4).

**Table 4:** Comparison of the mean C_pwr_, axis, and Jackson cross-cylinder power in the subgroups for C_pwr_

Subgroup for C_pwr_	Age (Year)	N	Topcon TRK-2P	Nidek Handy Ref-K	*P*-value
	Mean (Range)		Mean ± SD		
C_pwr_ ≥ − 2.00	6.2 (5–13)	64			
C_pwr_ (D)			−2.42 ± 0.95	− 2.49 ± 0.97	0.245
C_ax_ (^0^)			106.11 ± 79	130.37 ± 68.52	0.262
J_0_ (D)			−0.26 ± 0.86	− 0.21 ± 0.80	0.831
J_45_ (D)			−0.26 ± 0.93	0.03 ± 1.1	0.447
−1.00D ≤ C_pwr_ < − 2.00	6.5 (5–14)	51			
C_pwr_ (D)			−1.13 ± 0.13	−1.05 ± 0.34	0.355
C_ax_ (^0^)			76.19 ± 76.71	78.1 ± 79.51	0.134
J_0_ (D)			−0.12 ± 0.30	−0.18 ± 0.38	0.709
J_45_ (D)			0.03 ± 0.48	−0.12 ± 0.36	0.351
C_pwr_ < −1.00D	7.9 (5–16)	141			
C_pwr_ (D)			−0.38 ± 0.25	−0.52 ± 0.34	**< 0.001**
C_ax_ (^0^)			79.22 ± 71.11	97.71 ± 73.74	**0.025**
J_0_ (D)			−0.04 ± 0.15	−0.02 ± 0.18	0.251
J_45_ (D)			−0.02 ± 0.17	−0.05 ± 0.24	0.171

*C*_*pwr*_ Cylindrical power, *C*_*ax*_ Cylindrical axes, *SD* Standard deviation, *D* Diopter, *J*_*0*_ Jackson cross-cylinder power at 0° axis, *J*_*45*_ Jackson cross-cylinder power at 45° axis

## Discussion

Our findings showed that in the early detection of amblyogenic refractive errors, two auto-refractometers might be used interchangeably in children who were capable of adequate cooperation during measurement. Additionally, in children who have poor collaboration during measurement, the Nidek HandyRef-K could be used instead of Topcon TRK-2P. In the subgroup analysis, the differences between the measurements of the two auto-refractometers were likely to be within clinically applicable limits though there were some minor differences.

Some studies have been reported the reliability and agreement limits of auto-refractometers. For example, Ying GS et al. [15] evaluated the agreement limit of a table-mounted and hand-held auto-refractometer and reported that mean differences and 95% LoA were 0.34 D (− 0.46 D to 1.14 D) for S_pwr_; 0.18 D (− 0.47 D to 0.64 D) for C_pwr_; 0.25 D (− 0.55 D to 1.05 D) for SE. They reported the proportion of differences within the accuracy of 0.50 D as 56.9% for S_pwr_ and 70.2% for SE. Additionally, Büchner TF et al. [16] reported the proportion of differences within the accuracy of 0.50 D as 18.2% for SE, 82.1 for C_pwr,_ and 66.6 for C_ax_.

Sayed KM et al. [17] compared table-mounted and hand-held auto-refractometer measurements and found strong positive correlations for S_pwr_ and C_pwr_. The hand-held auto-refractometer measured more myopia regarding SE. They reported good agreement limits for C_pwr_ despite the relatively poor agreement limits for SE, J_0,_ and J_45_. Iuorno JD et al. [18] also reported that a hand-held auto-refractometer measured more myopia than a table-mounted auto-refractometer regarding SE though it had reliable results for C_pwr_.

The accuracy of auto-refractometers’ measurements of S_pwr_, C_pwr_, SE, and C_ax,_ varies depending on cycloplegia. Mirzajani et al. [19] reported prominent variation in the S_pwr_, SE, and J_45_ vector between a table-mounted and a hand-held auto refractometer in non-cycloplegic condition. These authors found a strong positive correlation and fair agreement for S_pwr_, SE, J_0_, and J_45_ vectors.

Akil et al. [10] compared outcomes of hand-held and table-mounted auto-refractometer. They evaluated significantly hyperopic results for mean SE with the table-mounted auto-refractometer before cycloplegia. Good agreement and no significant differences were obtained for S_pwr_, C_pwr_, J_0_, and J_45_ among two devices and cycloplegic retinoscopy after cycloplegia.

In a cross-sectional study, Oral et al. [20] evaluated the cycloplegic results of a hand-held autorefractor with cycloplegic retinoscopy and reported no significant difference in terms of mean S_pwr_, C_pwr_, and SE, and a strong correlation among devices.

Farook et al. [21] compared a hand-held autorefractor with a table-mounted autorefractor and subjective refraction. They found that the hand-held autorefractor measured more myopia than the table-mounted autorefractor and subjective refraction. However, their measurements were in non-cycloplegic condition and included only adult participants.

Seymen et al. [22] compared three hand-held autorefractors (HandyRef-K, Retinomax, and Plusoptix). These authors reported no significant difference among the three hand-held devices for the mean S_pwr_ and C_ax_. However, the mean SE measured with Plusoptix was significantly more myopic compared to those measured with the HandyRef-K and Retinomax devices. The authors also found that the mean C_pwr_ measured by the HandyRef-K device was considerably higher compared to Plusoptix and Retinomax. In their study, refractive measurements with the Plusoptix device were taken in non-cycloplegic conditions, while those with HandyRef-K and Retinomax were in cycloplegic states. Moreover, these authors did not compare the mean J_0_ and J_45_ values.

Astigmatism is a significant amblyogenic factor. Yap et al. [23] showed that lower magnitudes of astigmatism could also cause amblyopia and meridional deficits in the visual cortex of the newly diagnosed meridional amblyopic patients. Some studies reported that prevalences of meridional amblyopia were 30, 35, and 63% in patients with high astigmatism [24, 25]. This current study showed that meridional amblyopia was present in only 14 (21.9%) of the 64 patients who had 2.0 D or more astigmatism. This relatively lower percentage may be related to the fact that most patients were not newly diagnosed and had been complying well with spectacles and patching treatment that prevented them from getting amblyopia. This study had some limitations. The primary flaw was not comparing the results with cycloplegic retinoscopy. Unfortunately, we could not measure cycloplegic retinoscopy from all patients due to technical problems with the device when the study continued and did not gather enough cycloplegic retinoscopy results for the comparison. We only compared the measurements of two devices with each other, not with the results of cycloplegic retinoscopy. Therefore this study could not determine which device was more accurate. We also did not compare the repeatability of S_pw_, C_pwr_, and C_ax_ with either device.

In conclusion, the two autorefractors showed clinically applicable agreement limits, high PPA within 0.50 D for S_pwr_, C_pwr_, SE, J_0_, and J_45,_ excellent reliability for S_pwr_ and SE, and good reliability for C_pwr_ in cycloplegic conditions, though the Nidek HandyRef-K measured more astigmatism and less hyperopia in comparing the mean C_pwr_ and SE, and there existed some minor differences in subgroup analysis. The results from this current study showed that both devices might be used interchangeably for making clinical decisions and pediatric refractive screening. These differences, agreement intervals, and reliability of two auto-refractometers should be kept in mind in clinical practice and national pediatric vision screening programs to correct the refractive error.

## Data Availability

All data generated or analyzed during this study are included in this published article.
